# Alhemo’s FDA approval: a new treatment option for Hemophilia A & B

**DOI:** 10.1097/MS9.0000000000003710

**Published:** 2025-08-20

**Authors:** Zain Ul Abideen Hanif, Huzaifa Ali, Mohib Ullah, Rafiullah Hotak

**Affiliations:** a Ayub Medical College, Abbottabad, Pakistan; b Faculty of Medicine, Nangarhar University, Jalalabad, Nangarhar, Afghanistan

*Dear Editor,*

In the pursuit for the betterment a milestone has been achieved by the recent FDA approval of Alhemo (concizumab) for the Hemophilia A and B.

Hemophilia A and B are rare X-linked recessive genetic disorders prevalent predominantly in men caused by mutations in the genes encoding clotting factor VIII (FVIII) and factor IX (FIX). It is characterized by impaired blood clotting leading to frequent bleeding episodes, bruising, joint pain and swelling, muscle pain and weakness, pallor, fatigue, shortness of breath etc. If left untreated, severe bleeding can lead to blood loss anemia, shock, organ failure and eventually death. Hemophilia A (HA) occurs more frequently than Hemophilia B (HB), with an incidence of 1 in every 5000 male births in contrast to 1 in 30 000 for HB. A total of 347 026 patients had been identified in a global survey in 126 countries in 2020^[1]^.

The new approval of Alhemo is considered to be a significant option for the better quality of life for patients by reducing the amount of infusion, Alhemo of the class monoclonal antibodies. This letter aims to address the basic benefits and inconveniences in terms of Alhemo’s approval by evaluating the efficiency, safety profile, limitations, adverse reactions and quality in terms of Alhemo as a new treatment option. This study was reported in accordance with the TITAN (Transparency in the Reporting of Artificial Intelligence) guideline to ensure clarity, transparency, and reproducibility in the use and reporting of AI-based methods^[2]^.

## Current treatment option

At present, there is a variety of treatment options available for Hemophilia A and B. Which primarily involves replacement therapies of factor VIII (FVIII) and factor IX (FIX), desmopressin (DDAVP), antifibrinolytic agents, bypassing Agents, gene Therapy, extended half-life (EHL), factor products, subcutaneous factor replacement^[3]^. In factor replacement therapy patients are given on demand treatments to prevent acute bleeding or prophylactically (regular infusions ⅔ weeks for factor VIII and once for factor I X). Antifibrinolytic drugs are used to stabilize clots by preventing their breakdown particularly for mouth bleeds and post-surgery. Bypassing agents are used for promoting clot in patients with inhibitors .Factor VIIa (NovoSeven) and activated prothrombin complex concentrate (FEIBA) are potential bypassing agents. Gene therapy emerging as a potential cure, copy of gene that codes for the factors VII and IX is introduced in the patient. Some of the new treatments like EHL aim to modify factors VII and IX to stay longer in body and reduce the number of infusions. Similarly subcutaneous factor replacement in which the factor replacement is administrated subcutaneously giving a more convenient mode of administration^[4]^. Although alleviative and effective these treatment options still show significant shortcomings to the challenges faced in management of hemophilia.

Some of the limitations posed by the current treatments include the frequent intravenous transfusion, venous complications, development of immune responses that develop antibodies (inhibitors) against factors VII and IX detrimental to replacement therapies, short half-life, high cost, and less availability^[4]^. The current treatment options only manage the conditions rather curing the disease. Hence the need of new treatment options that can address suitable dosing regimens, reduce inhibitor formation, and increase portability, convenience, and overall quality of life. Approval of Alhemo is believed to resolve some of these gaps and shortcomings (see Table 1).

**Table 1:** The summary of FDA-approved drugs for Hemophilia A and B

Drug	Date of approval	Class	Mode of administration	Clinical trials
Roctavian	29 June 2023	Gene therapy	Single dose intravenous infusion	Multinational study in adult men 18–70 years old with severe Hemophilia A; 134 men enrolled in the GENEr8-1 study
Emicizumab	16 November 2017	Hemostatic antibodies	Subcutaneous injection, once weekly	Tested in adults and children, ages newborn and older, with hemophilia A with or without factor VIII inhibitors ^1^
Desmopressin	1978 (initial approval)	Synthetic antidiuretic hormone, vasopressin analog	Intravenous, subcutaneous, or intranasal administration	Tested in patients with diabetes insipidus, hemophilia A, and von Willebrand disease
Aminocaproic acid	1964 (initial approval)	Antifibrinolytic agent	Oral or intravenous administration	Tested in patients with hemophilia, hereditary angioedema, and to reduce bleeding after surgery or trauma
Sevenfact	1 April 2020	Coagulation factor VIIa concentrate, recombinant	Intravenous injection	PERSEPT 1, a multicenter, open-label trial evaluating 27 adult and adolescent hemophilia A or B patients with inhibitors, with 468 mild, moderate, and severe bleeding episodes treated

## Alhemo: a promising treatment option for Hemophilia A and B

Alhemo is an antagonist of tissue factor pathway inhibitor (TFPI) that is approved for use as routine preventative treatment to decrease the occurrence of bleeding episodes in both adult and pediatric patients aged 12 years and older with Hemophilia A and B^[5]^.

### Dosage and administration^[5]^:

Alhemo is administered subcutaneously mainly in the abdomen or thigh (recommended to rotate injection sites on alternate days)

Alhemo is administered in two phases:

1. Loading phase: Only the first day of administration is the loading phase where patient is given a loading dose of 1 mg/kg of their body weight.
2. Maintenance phase: The second day and onwards counts the maintenance dose of Alhemo which is 0.2 mg/kg of patient’s body weight. Maintenance phase ends with individualization of the maintenance dose 4–8 weeks after the start of the treatment.
3. Four weeks following the start of treatment: To optimize the dose, assess the plasma concentration of concizumab-mtci using the Concizumab Enzyme-Linked Immunosorbent Assay (ELISA) before the next scheduled dose is given.
4. Once the results for concizumab-mtci concentration are received, tailor the maintenance dose of Alhemo within 8 weeks of starting treatment, guided by the following plasma concentration levels of concizumab-mtci:

[A] Below 200 ng/mL: modify to a daily dose of 0.25 mg/kg.

[B] Ranging from 200 to 4000 ng/mL: maintain the daily dose of 0.2 mg/kg.

[C] Exceeding 4000 ng/mL: change to a daily dose of 0.15 mg/kg.

## Clinical trials

Clinical trials of Alhemo conducted by Novo Nordisk to estimate the efficiency and safety of Alhemo in adults and adolescents older than 12 years. Hence a multi-center open label non randomized phase 3 trial was conducted on 133 patients among which there were 92 adult and 42 adolescent patients (NCT04083781). Initially phase 1/2 trial (explorer 1) was conducted to evaluate Alhemo in healthy volunteers, explorer 2, 3, and 4 of the phase two trial in patients with Hemophilia A and B with and without inhibitors and in pediatric patients respectively. In phase 3 (explorer 5 6 7) Alhemo was assessed in patients with Hemophilia A and B with and without inhibitors. In explorer 7, of the 133 patients 52 males were randomly assigned among which patients were divided in arm 1 given no prophylaxis (n = 19) and arm 2 given Alhemo prophylaxis (n = 33), the other 81 males were non randomly assigned and divided into arm 3 and 4 receiving Alhemo prophylaxis. Alhemo was administered subcutaneously via a prefilled, premixed pen in the patients initially with a dosage of 1 mg/kg of body weight and continuing 0.2 mg/kg daily. After 24 or 32 weeks of completion primary endpoint was recorded as Annualized bleeding rate (ABR) in between arm 1 and arm 2 and supportive secondary endpoints constituting proportion of patients with zero bleeds. Quality of life assessments, and safety evaluations, to find the optimal dosage dose-titration regimen, a 12 month of treatment period with Alhemo under regular assessments was conducted. Patients who completed their treatment period were allowed to enter the extensive period^[6]^.

Explorer 7 study results showed 86% reduction of spontaneous and traumatic bleeds in patients practicing Alhemo. Patients using Alhemo prophylaxis were found to have an estimated mean ABR of 1.7 in comparison to the 11.8 estimated ABR of patients who received no prophylaxis. Among the patients on Alhemo prophylaxis 64% patients experienced zero spontaneous and traumatic bleeding during the first 24 weeks.18 adverse reactions in 14 patients including one hypersensitivity reaction and one thromboembolic event both constituting for 0.9 % each. In 26 patients constituting 22.8% injection site reactions were reported in which 7.9% injection site erythema and 3.5% injection site bruising along with one moderate injection site rash occurred due to Alhemo treatment. Similarly increasing levels of fibrin D-dimer were reported in 6 (5.3%) of patients and increased levels of fragment 1.2 were seen in 7 (6.1%) patients. These trials formed the basis for the approval of Alhemo^[7]^.

## Limitations

The study of Alhemo offers important insights; however, the clinical trials supporting approval were conducted on a restricted study population, excluding key subgroups such as patients with comorbidities and pediatric populations. This raises questions about the drug’s safety and efficacy in real-world, diverse patient cohorts. Additionally, the approval was granted based on short-term efficacy data, leaving uncertainties about long-term outcomes, durability, and potential adverse effects. Moreover, the trials featured a small sample size, which limits statistical power and fails to capture rare side effects. Alhemo is specifically indicated for patients with Hemophilia A or B who have developed inhibitors against factor VIII or IX. Injection site reactions and hives (urticaria) are potential adverse effects^[5]^ of Alhemo delivered via subcutaneous, intramuscular, or intravenous injection. The prescribing information recommends measuring concizumab-mtci plasma concentrations for dose optimization

Alhemo is currently approved only for patients aged 12 years and older

The lack of comparative data with existing treatments further complicates decision-making for clinicians seeking the most effective therapy for their patients.

## Recommendations

This endorsement represents a major advancement in hematological research and has the potential to significantly improve the lives of individuals affected by Hemophilia A or B. It is essential that healthcare professionals across various fields receive timely information about Alhemo, so they understand it as a legitimate treatment option. Given its promising effectiveness, it is vital that this medication is made widely available to assist as many patients as possible. Strategies regarding pricing must prioritize affordability and fair access, particularly in areas with limited resources. Partnerships with patient advocacy organizations will play a key role in increasing awareness, encouraging early diagnosis, and helping patients obtain treatment.

Moreover, treatment protocols and policies should be revised to incorporate Alhemo as a standard treatment option. In addition, training sessions and workshops ought to be organized for healthcare professionals to educate them on the administration and management of Alhemo. Initiatives aimed to promote adherence and enhance outcomes. Nonetheless, while this approval marks a significant achievement, it is vital to continue investing in research and development to improve treatment results for individuals with Hemophilia A or B, constantly aiming for enhanced patient care. Ongoing monitoring and real-world studies will be essential to assess long-term efficacy and safety, making sure that the benefits observed in clinical trials are successfully replicated in everyday clinical settings.

## Conclusion

Hemophilia A and B is a genetic disorder which causes inability to clot and spontaneous bleeding. Although there are numerous medications until now which treated the symptoms rather than the underlying conditions, Alhemo represents a major advancement by targeting the underlying mechanism. FDA approval of Alhemo is believed to overcome some of the gaps and shortcomings of the previous treatments. Even after its approval it is yet to be seen that will it prove revolutionary in terms of managing hemophilia. Further studies trials and evaluation should be done because many aspects of the drugs efficiency, adverse reactions, and reliability are yet unknown. Along with it Alhemo should also be assessed in the wider population in the different regions of the world to estimate its universality.

## Data Availability

Not applicable.
